# Experimental investigation on the ultrasonic impregnation of wood through measurements of the intensity of sonoluminescence

**DOI:** 10.1016/j.ultsonch.2022.106084

**Published:** 2022-07-03

**Authors:** Toru Tuziuti, Kyuichi Yasui

**Affiliations:** National Institute of Advanced Industrial Science and Technology (AIST), Moriyama, Nagoya 463-8560, Japan

**Keywords:** Cavitation bubble, Amplitude, Surface, Immersion

## Abstract

Ultrasonic impregnation is thought to be an effective way of permeation of liquid into material through the material-surface reforming with the attack by an ultrasonic cavitation jet or by the shock wave emitted from a collapsing bubble, or through dynamic transformation of material like a sponge. The action of a cavitation bubble can also provide penetration of liquid into the interior of the material. This paper investigates whether there is a correlation between the intensity of sonoluminescence (SL) measured at different positions and the increment in the mass of the wood material (cedar) after sonication with immersion into water in order to clarify the role of cavitation bubbles for ultrasonic impregnation. It was found that a high mass change was obtained for the material located at the position for high (the maximum) SL intensity. The number density of ultrasonic cavitation bubbles that are able to collapse leading to the emission of SL is correlated with the degree of ultrasonic impregnation.

## Highlights

• Sonoluminescence (SL) intensity in the vicinity of wood material was measured.
• High relative mass change per input power was obtained at high SL intensity.
• The number density of ultrasonic cavitation bubbles responsible for SL at the collapse is correlated with the degree of ultrasonic impregnation.

## 1 Introduction

Ultrasonic impregnation of media into wood material is thought to be from the extrusion of an air layer from inside the material [1]. The literature implies that the energy of a cavitation bubble released with its expansion and contraction has the potential to cause an effective transformation of the air layer to different media promoting dissolution, emulsification, and dispersion. Zhao et al. [2] claim on the role of ultrasonic cavitation bubbles that an ultrasound propagating in a liquid causes a shock wave and the shock wave caused by an ultrasonic cavitation bubble at collapse promotes extrusion of an extract sticking to the cell walls of wood to increase the permeability of the wood and mass transfer, and therefore, the efficiency in the impregnation is expected to increase. Amemiya and Siriban set simultaneously three pieces of wood plates at different positions from a transducer and sonicated [3]. They showed that promotion of permeation of water into a plate was obtained for the one closest to the transducer. Lin et al. [4] compared the ability to impregnate an antifungal agent into wood between sonication and vacuum. They showed that although both provided the effect of an antifungal agent, the extent of the effect with sonication was low compared with that with vacuum. Tsuruta impregnated chemicals into wood material by pressing a horn-type transducer driven at 18 kHz into the material [5]. Zhao et al. [2] and Tsuruta [5] implied that cavitation could be related to impregnation; however, their evidence was not identified. So far, little is known about attempts like the present investigation to clarify a mechanism of ultrasonic impregnation of wood material by paying attention to the phenomena of sonoluminescence. Sonoluminescence is the light emission phenomenon from collapsing bubbles in liquid irradiated by an ultrasonic wave [6], [7], [8]. Sonoluminescence is able to provide information on number of active cavitation bubbles [9], [10], [11].

To the best of the authors’ knowledge, it does not seem to be necessarily clear whether an ultrasonic cavitation bubble is able to contribute to impregnation and also whether the optimized sonication condition for cavitation bubbles to enhance efficiency in impregnation has been established. This paper clarifies the role of cavitation bubbles on ultrasonic impregnation through measurements of intensity of sonoluminescence and relative mass change between before and after an immersion process including ultrasonic impregnation.

As for the wood material for ultrasonic impregnation, beech was used in the literature [1]. In the present experiment, cedar is used. This is because cedar can be obtained easily. Cedar is used widely as a building material for houses in Japan. According to the literature [1], there are advantages including not only high-efficient homogeneous impregnation of liquid into material but also an inexpensive apparatus when using ultrasonic impregnation in comparison with the other methods (the decompression impregnation method and the decompression-pressurization impregnation method).

## 2 Materials and methods

**Fig. 1:**
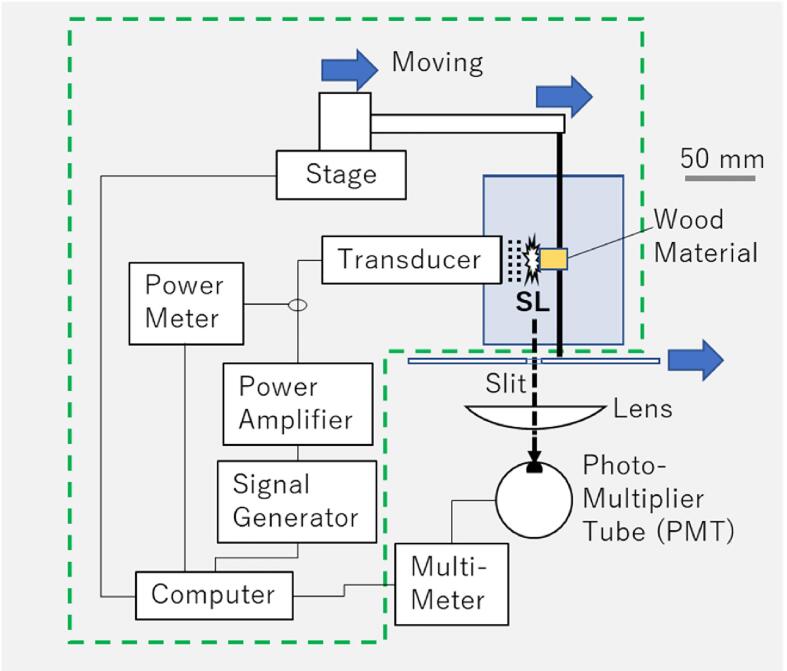
Schematic of the apparatus for the intensity measurements of SL. The region surrounded with a dashed line frame was used for the ultrasonic impregnation experiment. The scale bar is for the configuration of the transducer, container and wood material.

Fig. 1 shows a schematic of the apparatus for the intensity measurements of SL. In a rectangular container of 100 × 120 × 120 mm^3^ inner dimensions filled with 1.2 L of air-saturated pure water (Millipore Essential Elix 5), sonication of 38-kHz continuous-wave sinusoidal ultrasound was performed from the container side driving a horn-type transducer of 33 mm in diameter toward the wood material (cedar) of 15 × 15 × 20 mm^3^. The temperature of the water was kept to be 22–23 °C using a thermostatic chamber (Fine FR-100) before sonication.

Calorimetric studies were done for near 7 mm and 27 mm for the wood material position. The signal amplitude was 300 mV (peak-to-trough). A sheath-type K thermocouple (ADVANTEST TR-1101–130) of 1.6 mm in diameter was set near the surface center of the wood material to determine the liquid temperature every 2.5 s while the sonication was applied for 5 min (300 s). A digital multimeter (ADVANTEST TR6847) was used for reading the temperature and the data were stored in a computer. The measurements were done three times for each position. Calorimetric power was calculated as the product of specific heat of water, mass of water, and temperature gradient during sonication [12].

The results showed that the calorimetric power measured was 10 W and 7.5 W near 7 mm and 27 mm for wood material position, respectively. As for the average electric input power, 14.6 W and 11.9 W were obtained for 7 mm and 27 mm for the wood material position, respectively. It was found that each of the powers was higher for the case of near 7 mm than for the 27 mm case. This implied that, for near 7 mm, heating of the wood material which absorbed sound energy rather than that through thermal conduction from the interior of cavitation bubble was able to cause a temperature rise in the liquid (water). The difference between the calorimetric and electric input powers for each of the positions probably comes from the dissipation of thermal energy towards the outside of the container.

The reason why there is the difference in the measured electric powers is as follows. When the wood material is closer to the transducer, the wood material is able to absorb more acoustic energy than when the position is farther from the transducer. In this situation, the component of the reflected wave back to the transducer in the standing wave between the transducer and the wood material becomes less and the electric power is easier to input to the transducer. In this background, the solid angle of wood material facing the transducer center in the former is larger than that in the latter.

The results of calorimetric studies show that temperature evolution per unit time was 2.0 × 10^-3^ °C/s and 1.5 × 10^-3^ °C/s near 7 mm and 27 mm for the wood material position, respectively. Since the sonication time was 300 s (5 min), temperature rise by sonication would have been less than 1 °C.

The mass of the wood material measured using an electronic balance (A&D HF-400) before immersion was 1.662 ± 0.037 g. The signal amplitude from a signal generator (NF 1946A) given to the power amplifier (ENI 1140LA) driving the transducer was a constant 300 mV (peak-to-trough) unless intentionally made to change to the different one. To evaluate the extent of cavitation around the wood material, SL intensity only from around the wood material was measured with a photomultiplier tube (PMT; Hamamatsu Photonics R928), a digital multimeter (ADVANTEST R6552) and the wood material far from the transducer with a slit of 20 × 12 mm^2^. The center position of the slit was set at the transducer surface in the direction normal to the sound propagation. The side wall of the container facing the PMT was made of glass to pass SL. The position of the wood material was changed along with the sound beam axis with a motorized moving stage (SIGMAKOKI OSMS20-85) controlled by a computer (DELL GX260), where both of the slit and the wood material connected with the stage moved together.

On evaluation for the increment in the mass of the wood material immersed in water after sonication (relative mass change) per unit input power to the transducer, the impregnation process was performed in the order of pre-immersion without sonication (5 min), sonication (5 min), and storage in a bottle filled with water after sonication (up to 110 min). Namely, the total immersion time was 120 min (2 h). Relative mass change was estimated using measured mass difference in the wood material between before and after the immersion process including 5-min sonication.

In order to investigate whether there is a correlation between relative mass change by sonication and SL intensity, relative mass change of the wood material was evaluated for the two positions showing the minimum and maximum for SL intensity (7 mm and 27 mm from the transducer, to be shown in later).

Input power to the transducer was measured with a power meter (TOWA ELECTRIC TAW-60A) in order to compare relative mass change per unit input power between different positions. The input power to the transducer measured in the present experiment was in the range between 11.5 W and 16.0 W, and the average was 13.3 W. The average acoustic intensity [13] in the present experiment was 1.56 W/cm^2^. This was lower than the 8 W/cm^2^ required for cone-like bubble formation in ultrasonic cavitation field shown in the literature [13]. This suggested that there occurred few cavitation bubble clouds in the vicinity of the surface of the transducer under the experimental conditions of the present study.

Sound pressure amplitude for the positions showing the minimum and maximum SL intensities was measured with a calibrated hydrophone (Brüel & Kjær 8103) to discuss the difference in bubble dynamics between the positions.

## 3 Results and discussion

**Fig. 2:**
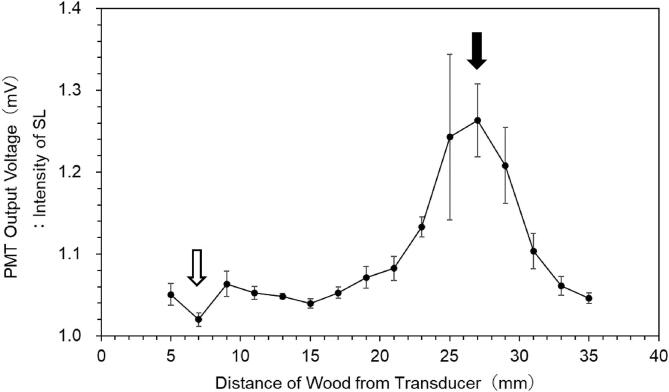
Dependence of the intensity of SL in the vicinity of the wood material on the distance from the transducer. Error bars represent the average relative error for the data measured five times at each distance. Open and filled arrows indicate the distances of wood from the transducer showing the minimum and the maximum SL intensities, respectively.

Fig. 2 shows the dependence of the intensity of SL around the wood material on the distance from the transducer. Almost 1 mV in PMT output voltage comes from the background of the experimental circumstance. It was found that SL intensity was not high closer to the sound source. Maximum intensity was obtained at the position distant from the sound source. This is consistent with the literature by Nomura and Nakagawa [9], including the evaluation of physical effects of an ultrasound [9], [10], [11]. In the literature [9], as Nomura and Nakagawa moved the aluminum foil attached on the surface of a block bottom distant from a transducer driven at 40 kHz, they measured the loss of foil in the sound field with a high enough amplitude to cause cavitation bubbles. They showed that the maximum loss was obtained at a position distant from the transducer.

Supposing the wood material will be relatively close to the transducer, the sound pressure amplitude between them will be high, while the sound pressure amplitude in the vicinity of the wood material will lower from damping of sound energy due to spatial dispersion as it gets farther from the transducer. Indeed, the hydrophone measurement of sound pressure amplitude showed that the sound pressure amplitude was 434 kPa when the wood material was positioned at 10 mm distant from the transducer, which was close to the position where the minimum SL intensity occurred (7 mm), while the sound pressure amplitude was 147 kPa at 27 mm, where the maximum SL intensity was obtained. Thus, it was confirmed that the latter sound pressure amplitude was lower than the former. Note here that the reason why not 7 mm but 10 mm was chosen for the position of the wood material was that the hydrophone, which had a size of a little larger than 7 mm, was impossible to insert within the span of 7 mm between the transducer and the wood material. According to the literature by Krefting et al. [7], a high-pressure amplitude region did not necessarily provide physical effects such as erosion [9], [10] due to the depletion of cavitation bubble nuclei from the region by the action of a repulsive primary Bjerknes force [10], [14]. Similarly, high-pressure amplitude in the vicinity of the transducer in the present experiment would expel some of the active cavitation bubbles.

Hatanaka et al. clarified that the expelling of cavitation bubbles from the region of high sound pressure amplitude results in a decrease in the intensity of SL [14]. This is the probable reason why the observed SL intensity was relatively low at 7 mm, where the wood material was close to the transducer. When the wood material was farther from the transducer, the sound pressure amplitude became lower, leading to the relaxation of the expelling of cavitation bubbles. The position 27 mm from the transducer, where the maximum intensity of SL was obtained, was rather close to the antinodal position (30 mm) of a standing wave with a span of three-fourth of the wavelength of the ultrasound between the transducer and the wood material. These aspects of the sound field resulted in the maximum intensity of SL observed near the wood material when the wood material was far from the transducer by a certain distance.

Lee et al. [15] captured SL images from air-saturated water in a sonicated vessel where the surface of the sonicated water was free (air) and the bottom was a plate connected to a transducer to show that no SL activity was near the bottom (transducer). They also calculated numerically the ratios of the standing- and progressive-wave components constituting the sound field to show that the ratio of the standing-wave component was higher than that of the progressive one near the water surface for a low ultrasonic frequency, while the former was lower than that of the latter near the transducer [16], [17]. These results by Lee et al. [15] were consistent with that, in the present experiment, efficient SL activity was obtained at around the antinode of sound pressure nearest to the boundary (the surface of the wood material) distant from the transducer.

Commercial software (Excel Microsoft 365) was used to statistically analyze the difference between the data for relative mass change. The software includes the statistical analysis of *t*-test. p-value is a value to judge whether there is a difference between two independent populations by comparing the value with a statistical significance level.

Table 1 shows raw mass change with immersion time at different positions of the wood material together with p-value for the difference. As the immersion time proceeded, raw mass change for both 7 mm and 27 mm increased. If a statistical significance level of 5% is chosen, each of the p-values of 10%, 39%, and 74% for 10 min, 1 h, and 2 h, respectively, is larger than 5% and the difference in the raw mass change between them is little. The raw mass change obtained at 27 mm comparable to that at 7 mm seems to be high considering low input power and farther position from the transducer. This probably comes from the action of cavitation bubbles.

**Table 1:** Raw mass change with immersion time at different positions of the wood material together with p-value for the difference.

immersion time	(1) raw mass change (g); at 7 mm	(2) raw mass change (g); at 27 mm	Absolute Difference (g); between (1), (2)	p-value (%)
10 min	1.893	1.816	0.077	10
60 min (1 h)	2.232	2.184	0.048	39
120 min (2 h)	2.256	2.235	0.021	74

Table 2 shows raw mass change with immersion time without/with sonication together with p-value for the difference. As the immersion time proceeded, raw mass change for both without and with sonication increased. It was found that raw mass change with sonication was higher than that without sonication for each of the immersion times. If a statistical significance level of 5% is chosen, each of the p-values of 2.2%, 4.4%, and 1.9% for 10 min, 1 h, and 2 h, respectively, is less than 5% and the difference in the raw mass change should be sufficient.

**Table 2:** Raw mass change with immersion time without/with sonication together with p-value for the difference.

(average of both distances) immersion time	(3) raw mass change (g); without sonication	(4) raw mass change (g); with sonication by 5 min	Difference (g); between (3) and (4)	p-value (%)
10 min	1.387	1.854	0.467	2.2
60 min (1 h)	1.830	2.208	0.378	4.4
120 min (2 h)	1.847	2.245	0.398	1.9

In order to investigate whether there is a correlation between mass change by sonication and SL intensity, mass change of the wood material by the penetration of the water was evaluated for the positions showing the minimum and maximum for SL intensity (7 mm and 27 mm from the transducer). Relative mass change was estimated using measured mass difference in the wood material between before and after the immersion process including 5-min sonication.

**Fig. 3:**
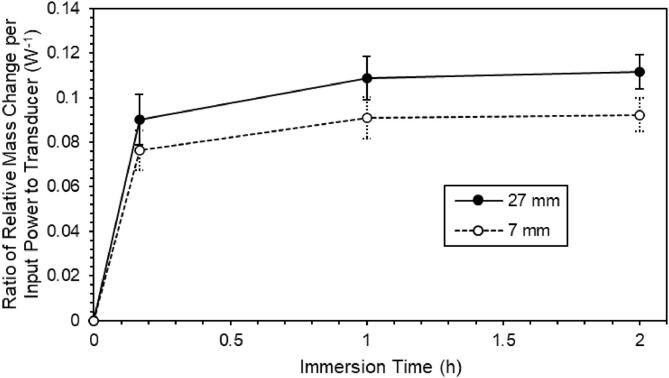
Ratio of relative mass change per input power to the transducer as a function of immersion time. Error bars represent the average relative error for the data measured five times at each of the immersion times.

Fig. 3 shows the ratio of relative mass change per input power to the transducer as a function of immersion time. The ratio of relative mass change per input power to the transducer, *r*, at 7 mm or 27 mm for the position of the wood material was defined as the following expression:

(1) $$r=\frac{\left(m_{a}-m_{b}\right)/m_{a}}{P}$$

where *m_b_* and *m_a_* were mass of wood material before and after immersion, respectively, and *P* was the input power to the transducer at 7 mm or 27 mm for the position of the wood material. As the immersion time proceeded, the ratio increased rapidly and, after that, saturated. It was found for each of the immersion times of 10 min (1/6h), 60 min (1 h), and 120 min (2 h) that a high mass change was obtained for the material located at the position (27 mm) for high (the maximum) SL intensity in Fig. 1.

Table 3 shows the average ratio of relative mass change per input power to the transducer with immersion time at different positions of the wood material together with p-value for the difference in the ratio. If a statistical significance level of 5% is chosen, each of the p-values of 3.2%, 1.7%, and 2.2% for 10 min, 1 h, and 2 h, respectively, is less than 5% and should be sufficient (Table 3). The obtained ratios at 27 mm were higher by 18%, 20%, and 22% after 10 min (1/6h), 60 min (1 h), and 120 min (2 h) of immersion time, respectively, compared with that at 7 mm.

**Table 3:** Average ratio of relative mass change per input power to the transducer with immersion time at different positions of the wood material together with p-value for the difference in the ratio.

immersion time	(a) ratio (1/W); at 7 mm	(b) ratio (1/W); at 27 mm	Difference between (a) and (b); [{(b) -(a)}/(a)] × 100%	p-value (%)
10 min	0.076	0.090	18	3.2
60 min (1 h)	0.091	0.109	20	1.7
120 min (2 h)	0.092	0.112	22	2.2

Note here that, compared with the mass change through the immersion process only (without sonication), it was confirmed that a high increment was obtained for the process including sonication. Table 4 shows the average of relative mass change with immersion time without/with sonication together with p-value for the difference in the increment. The obtained increments with sonication were higher by 36%, 23%, and 24% after 10 min (1/6h), 60 min (1 h), and 120 min (2 h) of immersion time, respectively, compared with that without sonication. As for the difference in relative mass change between the presence and absence of the sonication process, each of the p-values of 2.4%, 4.4%, and 2.1% is less than 5% and should also be sufficient.

**Table 4:** Average of relative mass change with immersion time without/with sonication together with p-value for the difference in the increment.

immersion time	(c) relative mass change; without sonication	(d) relative mass change; with 5-min sonication	Difference between (c) and (d); [{(d) -(c)}/(c)] × 100%	p-value (%)
10 min	0.824	1.124	36	2.4
60 min (1 h)	1.085	1.337	23	4.4
120 min (2 h)	1.095	1.359	24	2.1

Measured intensity of SL implies that an optimal configuration is required for an increase in the number density of spherical collapsing bubbles. Increase in the number density of spherical collapsing bubbles emitting shock waves effective for making a rough surface on the wood material can contribute to the removal of substances sticking to the surface of the wood material leading to promotion of impregnation of water into the wood material.

**Fig. 4:**
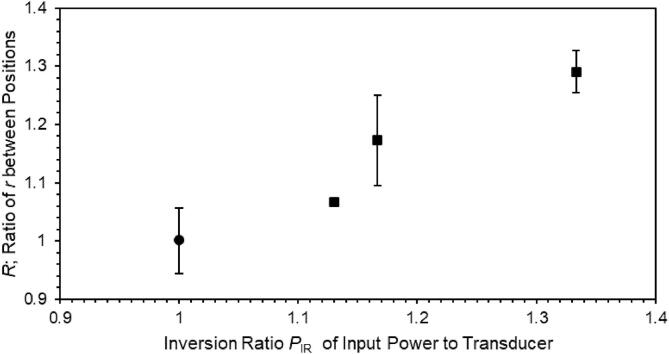
Average ratio *R* calculated by *r* (the ratio of relative mass change per input power to the transducer) at 27 mm divided by that at 7 mm for the positions of the wood material over all of the immersion process for 10 min, 1 h, and 2 h as a function of inversion ratio *P*_IR_ of input power to the transducer. Filled squares were for the signal amplitude which was a constant 300 mV (peak-to-trough). When the input power applied was selected to be the same between 7 mm and 27 mm by adjusting the signal amplitude, the inversion ratio was 1 (filled circle). An error bar indicates the average relative error.

The difference in the relative mass change between positions for the wood material was examined as follows. Fig. 4 shows the average ratio of *r* (the ratio of relative mass change per input power to the transducer) between 27 mm and 7 mm for the positions of the wood material over all of the immersion process for 10 min, 1 h, and 2 h as a function of the inversion ratio of input power to the transducer. The ratio of *r* between 27 mm and 7 mm for the positions of wood material, *R*, was defined as the following expression:

(2) $$R=\frac{r_{27\;mm}}{r_{7\;mm}},$$

where *r*_27 mm_ was the ratio of *r* at 27 mm and *r*_7 mm_ was that at 7 mm. The inversion ratio of input power to the transducer, *P*_IR_, was defined as the following expression:

(3) $$P_{IR}={\left(\frac{P_{27\;mm}}{P_{7\;mm}}\right)}^{-1},$$

where *P*_27 mm_ was the input power to the transducer at 27 mm for the position of the wood material and *P*_7 mm_ was that at 7 mm.

What is the physical meaning of *P*_IR_? Larger *P*_IR_ is established when *P*_7 mm_ is larger and/or *P*_27 mm_ is smaller. In this situation, cavitation bubbles are permitted to stay around the wood material at 27 mm and are able to emit SL of higher intensity there since the sound pressure amplitude there is relatively low compared with that at 7 mm in the present experiment. Also at larger *P*_IR_, the results shown in Fig. 4 mean that relative mass change per input power when the wood material is 27 mm is high compared with that at 7 mm. On the other hand, when the wood material is near 7 mm, most of the cavitation bubbles are expelled by a high-pressure amplitude and able to emit SL of little intensity there. Therefore, the physical meaning of *P*_IR_ is a parameter related with the sound pressure amplitude having an influence on the number of cavitation bubbles responsible for relative mass change and the intensity of sonoluminescence in the presence of the wood material.

As *P*_IR_ increased, *R* increased. This implies that, even a not so high amplitude was able to provide relative mass change, and therefore, effective sonication is plausible for impregnation on a large amount of wood material arranged at the position far from the transducer. Note here that, in the present experiment, even when the signal amplitude to the amplifier to drive the transducer was the same 300 mV (peak-to-trough) (data when the inversion ratio is more than 1 in Fig. 4), the input power to the transducer was rather smaller when the wood position was far (27 mm) from the transducer compared with the near position (7 mm), while the data when the inversion ratio is 1 were the results obtained by adjusting the signal amplitude to maintain the same input power.

On the extent of contribution to impregnation through sonication, which of the attack by cavitation microjet [18], [19], [20], the motion of squeeze and release like a sponge (sponge effect) [21], [22], shock waves [2], [18], [19], [20] probably provided from the present spherical collapsing bubbles, or an unknown factor is effective is left for future study.

## 4 Conclusions

Through the measurements of sonoluminescence near the wood material and the relative mass change through the immersion process including sonication, it was clarified that the number density of ultrasonic cavitation bubbles that were able to collapse leading to the emission of SL was correlated with the degree of ultrasonic impregnation. Even a time span for sonication among the total time for the immersion process which was relatively short was able to provide relative mass change.

## Declaration of Competing Interest

The authors declare that they have no known competing financial interests or personal relationships that could have appeared to influence the work reported in this paper.
